# The Novel Zoonotic Pathogen, *Anaplasma capra*, Infects Human Erythrocytes, HL-60, and TF-1 Cells *In Vitro*

**DOI:** 10.3390/pathogens10050600

**Published:** 2021-05-14

**Authors:** Yongshuai Peng, Chenyang Lu, Yaqun Yan, Jinxing Song, Zhiyang Pei, Pihong Gong, Rongjun Wang, Longxian Zhang, Fuchun Jian, Changshen Ning

**Affiliations:** 1 College of Veterinary Medicine, Henan University of Animal Husbandry and Economy, Zhengzhou 450046, China; yongshuaipeng@hnuahe.edu.cn; 2 College of Veterinary Medicine, Henan Agricultural University, Zhengzhou 450046, China; luchenyang1997@163.com (C.L.); yanyaqun@163.com (Y.Y.); songjx1113@163.com (J.S.); wrj-1978@henau.edu.cn (R.W.); zhanglx8999@henau.edu.cn (L.Z.); 3 College of Animal Science, Tarim University, Alar 843300, China; peizhiyang777@163.com (Z.P.); gph918743836@163.com (P.G.)

**Keywords:** *Anaplasma capra*, zoonotic pathogen, erythrocyte, HL-60, TF-1

## Abstract

*Anaplasma capra*, a species of the family Anaplasmataceae, is zoonotic tick-borne obligate intracellular bacteria. There have been no reports of human infection with this pathogen since 2015. Therefore, the zoonotic characteristics of *A. capra* need to be further studied. To verify the ability of *A. capra* to infect human cells, *A. capra* were inoculated in human erythrocytes, HL-60, and TF-1 cell lines *in vitro*. Cell smears were taken after inoculation, using Giemsa staining, transmission electron microscope (TEM), chromogenic in situ hybridization and immunocytochemistry for detection. In the Giemsa staining, many dark colored corpuscles or purple granules were seen in the inoculated erythrocytes, HL-60, and TF-1 cells. The results of chromogenic in situ hybridization show that there were brown precipitates on the surface of most erythrocytes. Immunocytochemistry results show many dark brown vacuolar structures or corpuscles in the cytoplasm of erythrocytes, HL-60, and TF-1 cell lines. The *A. capra* morulae were seen in the cytoplasm of both HL-60 and TF-1 in TEM, and their diameter was about 295–518 nm. Both dense-cored (DC) and reticulate cell (RC) form morulae could be seen. This study confirmed the ability of *A. capra* to infect human erythrocytes, HL-60, and TF-1. This study is of profound significance in further verifying the zoonotic characteristics of the pathogen and for establishing an *in vitro* cultivation model.

## 1. Introduction

*Anaplasma capra* is one of the species of the *Anaplasma* genus that are now considered significant zoonotic pathogens [1]. As early as 2010, Zhou et al. (2010) [2] detected an unknown *Anaplasma* species in goat blood samples in the Chongqing region of China. In 2015, Li et al. [3] detected the above pathogen at the Mudanjiang Central Hospital in Heilongjiang in the blood of patients with a tick bite history. The infected patients primarily manifested fever, headache, fatigue, dizziness, and chills. After phylogenetic analysis based on 16S *rRNA*, *gltA*, *msp2*, and *msp4* loci, the above species was considered a new species of the *Anaplasma* genus; it was temporarily named *A. capra*. Similar to *A. phagocytophilum* and *A. ovis*, it could infect humans [4,5]. Moreover, it is similar to *A. ovis*, *A. centrale*, and *A. marginale* in being infectious to animal erythrocytes [6]. Over the past two decades, diseases caused by *Anaplasma* have spread rapidly among humans. Since the first report of human infection with *A. phagocytophilum*, a growing number of tick-borne pathogens with zoonotic potential have been discovered. They cause anaplasmosis or related diseases and pose a considerable impact on public health worldwide [7].

According to relevant reports from China, *A. capra* (initially considered *A. centrale*) can also infect cattle [2]. Nevertheless, the pathogen was named after the first discovered host, the goat [2]. In Heilongjiang, China, *A. capra* was first detected from *Ixodes persulcatus*, which was later regarded as its main transmission vector [3]. Besides, the DNA of this pathogen was also detected in several other tick species such as *Haemaphysalis longicornis*, *H. qinghaiensis*, *Rhipicephalus microplus*, *Dermacentor nuttalli*, and *D. abaensis* [8,9,10]. Therefore, these species could be the main carriers that spread the pathogen to humans and vertebrates by sucking blood. There is little information about the life history and reservoir host of *A. capra* in nature. Although different strains of *A. capra* have been detected in ticks and animals such as goats, cattle, and deer in various regions of the world [6,7,11,12], there is no evidence thus far that any form of the pathogen has been detected by microscope in the human blood cells. Thus, morphological investigation of *A. capra* in infected human blood cells plays an important role in guiding the laboratory examination of the pathogen from human samples. This is of profound significance to the diagnosis and treatment of anaplasmosis.

Since the successful *in vitro* cultivation of *Anaplasma phagocytophilum*, the technologies for the *in vitro* cultivation and isolation of other *Anaplasma* species have been developing rapidly [13]. *A. phagocytophilum* can form vacuoles in the cytoplasm of the host cells and replicate inside these vacuoles. In laboratory settings, the human promyelocytic cell line HL-60 is the most common cellular model for phagocyte research as it expresses the receptors to be invaded by the bacteria [14]. During intracellular growth, *Anaplasma* species transforms from a dense-cored (DC) form capable of invading the host cells to a reticulate cell (RC) form that replicates inside vacuoles [15]. Human-derived *A. capra* has been cultured in HL-60 cells. Although *A. phagocytophilum*, *A. ovis*, and *A. marginale* can all be cultured in the tick cell lines ISE6 and IDE8 [16,17,18], these cell lines are not easily storable, and, hence, not widely used in laboratory settings. All these studies provide excellent references for the *in vitro* cultivation of *A. capra*. TF-1 is an erythroleukemia cell line derived from human bone marrow, and its surface has receptors resembling that of erythrocytes. This was established by Kitamura et al. in 1987 [19]. We recently reported that *A. capra* could infect goat erythrocytes [20]. To verify the ability of *A. capra* to infect human cells, *A. capra* were inoculated in human erythrocytes, HL-60, and TF-1 cell lines *in vitro*. This research is of profound significance for further verifying the pathogen’s zoonotic characteristics and establishing an *in vitro* cultivation model.

## 2. Materials and Methods

### 2.1. Pathogen Collection and Identification

Initially, 5 mL of *A. capra*-positive goat venous blood with anticoagulant were collected. The erythrocytes were then isolated from the whole blood sample as per the erythrocyte/leukocyte isolation kit instructions (Haoyang Biotechnology, Tianjing, Hebei, China). Next, the isolated erythrocytes were resuspended in a double volume of Alsever’s solution (Solarbio, Beijing, China) and stored at 4 °C for subsequent use. Later, a four-fold volume of pre-cooled (4 °C) erythrocyte lysis buffer (Solarbio, Beijing, China) was added to the erythrocyte suspension by gentle pipetting to ensure uniform mixing; this suspension was then placed in a 4 °C refrigerator for 10 min until complete lysis of the erythrocytes was attained. Finally, the pathogen was collected by high-speed centrifugation (Beckman JXN-30, Miami, FL, USA) for 30 min at 30,000× *g* and 4 °C. This was followed by resuspension of the collected pathogen in 1 mL of the RPMI 1640 medium (HyClone, Logan, UT, USA) containing 10% FBS (Gibco, Carlsbad, CA, USA).

DNA was extracted from 300 µL of the pathogen suspension by using a bacterial genomic DNA extraction kit (LifeFeng Biotechnology, Shanghai, China) as per the instructions and stored at −20 °C for subsequent use. Nested PCR reactions targeting the *gltA* gene and conventional PCR targeting the heat shock protein (*groEL*) gene and the major surface protein4 gene (*msp4*) of *A. capra* were performed as previously described for *A. capra* identification [3].

### 2.2. Human Erythrocytes Isolation and Preserved In Vitro

After collecting 2 mL of human venous blood in an EDTA-Na_2_-coated blood collection tube, human erythrocytes were isolated with the goat erythrocyte isolation kit (Haoyang Biotechnology, Hebei, China) as per its instructions. The human erythrocytes were transferred to the RPMI 1640 medium (500 mL, HyClone, Logan, UT, USA) containing 10% FBS (50 mL, Gibco, Carlsbad, CA, USA); the cell density in the medium was adjusted to around 3 × 10^10^ cells/mL. They were then preserved in a CO_2_ incubator (3111, Thermo, Waltham, MA, USA) set at 37 °C with 5% CO_2_.

### 2.3. Thawing and Cultivation of HL-60 and TF-1 Cells

HL-60 and TF-1 cells were thawed and cultured by the same methods. Specifically, cell cryopreservation tubes were removed from the liquid nitrogen container, placed quickly into a 36–37 °C water bath, and shaken regularly to allow rapid thawing; this exercise was completed within 30–60 s. After wiping and disinfecting with 75% ethanol, the cryopreservation tubes were opened. The cell suspensions were pipetted into the centrifuge tubes dropwise with 10 mL of the culture medium and centrifuged at low speeds (500–1000× *g*) for 5 min. The supernatants were discarded, and the remaining cells were washed again with the culture medium. After proper dilution with the culture medium, the cells were placed in culture flasks and kept in a 5% CO_2_ incubator at 37 °C. The next day, the culture medium was replaced, and the cultivation was continued. Cells were subcultured when the confluence reached 90%.

### 2.4. Pathogen Inoculation

Each 500 µL of the identified *A. capra* suspension were inoculated into the human erythrocytes, HL-60, and TF-1 cells that were cultured *in vitro*. After inoculation, the pathogen cell suspensions were divided into twelve aliquots and cultured in 12-well plates. Meanwhile, the uninoculated cells were cultured as three negative controls. After 96 h of the normal *in vitro* cultivation of the inoculated cells, the samples from three wells were collected to examine the cell infection.

### 2.5. Cell Infection Identification

#### 2.5.1. Wright–Giemsa Staining

Cell smears were prepared from the uninoculated and inoculated human erythrocytes, HL-60, and TF-1 cells that were collected at different time points and examined by Wright–Giemsa staining.

#### 2.5.2. Immunocytochemistry

Cell smears were prepared from the uninoculated and inoculated human erythrocytes, HL-60, and TF-1 cells that were collected at different time points. The *A. capra*-positive serum samples collected from the goat were used as the primary antibody and incubated at 4 °C overnight. Subsequently, HRP-conjugated Rabbit Anti-Goat IgG (H + L) (Servicebio, Wuhan, China) was applied to the smears and incubated at 37 °C for 1 h. The antibody was visualized using 3,3′-diaminobenzidine (Servicebio, Wuhan, China), and the images recorded using a light microscope (Nikon, Tokyo, Japan). As negative controls, the antiserum from the *A. capra*-negative goat and *A. capra*-negative blood smears were processed in the same manner and examined.

#### 2.5.3. Chromogenic In Situ Hybridization (CISH)

Cell smears were prepared from the uninoculated and inoculated human erythrocytes collected at different time points. CISH was performed with a commercial kit according to the manufacturer’s protocol (Invitrogen, Carlsbad, CA, USA). Briefly, the blood films were reverse-stained with eosin, visualized using Digital Slice Scanner (Pannoramic MIDI, Budapest, Hungary), and fixed with a fixative (Solarbio, Beijing, China). The CISH probes were designed specifically for the *A. capra groEL* gene (KM206273). To obtain sufficient signal intensity, 16 probes were designed along the lncRNA/mRNA sequence of the *groEL* gene, which covered the entire fragment length of the RNA molecules. The probes were labeled with digoxigenin (DIG) at both ends, and the samples under test were considered positive when a brown precipitate was observed.

#### 2.5.4. Transmission Electron Microscopy (TEM)

The collected HL-60 and TF-1 cells were infiltrated with acetone and Pon 812 epoxy resin (SPI, West Chester, USA) (1/1, 2 h; then, 1/2, 12 h) and cured at 60 °C for 48 h. The cured resin blocks were trimmed, thin-sectioned, thin sections collected on formvar copper 200 mesh grids, and then post-stained with 2% aqueous uranyl acetate and Reynold’s lead citrate. The sections were examined using a HT7700 electron microscope (HITACHI, Tokyo, Japan).

## 3. Results

### 3.1. Wright–Giemsa Staining Results

**Figure 1:**
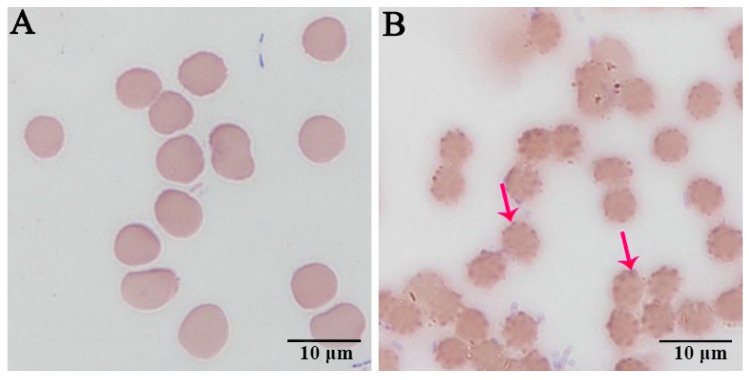
Wright–Giemsa-stained human erythrocytes: (**A**) an uninoculated human erythrocyte smear; and (**B**) the erythrocyte smear inoculated and infected with *A. capra*. The red arrows indicate *A. capra*.

**Figure 2:**
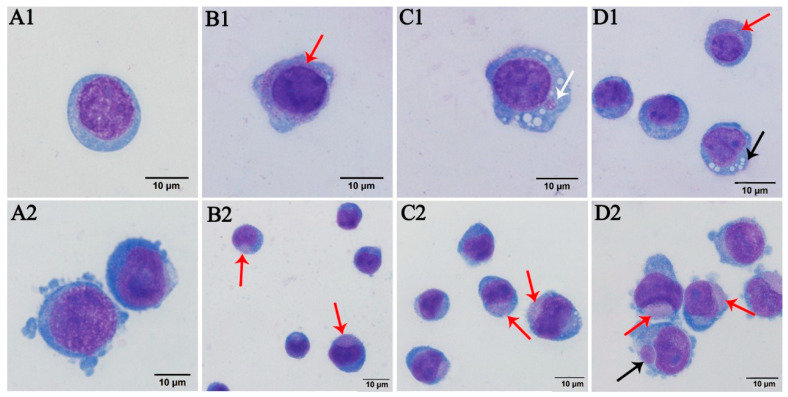
Wright–Giemsa-stained HL-60 and TF-1 cells, where the arrows indicate positive HL-60 and TF-1 cells: (**A1**,**A2**) the negative controls; (**B1**–**D1**) the *A. capra*-positive HL-60 cells, where the white arrow indicates a morula of *A. capra* and the black arrow indicates vacuole formation in the cytoplasm of the pathogen; and (**B2**–**D2**) *A. capra*-positive TF-1 cells, where the black arrow indicates a morula of *A. capra* and the red arrows in the figures indicate *A. capra.*

The human erythrocytes, HL-60, and TF-1 cells, which had been inoculated for 96 h, were subjected to Wright–Giemsa staining. Many darker stained bodies were visible and primarily distributed at the edges of the inoculated human erythrocytes (Figure 1B). In contrast, the edges of the uninoculated human erythrocytes did not exhibit this phenomenon (Figure 1A). Substantial purple-reddish granules were observed inside the HL-60 cells (Figure 2B1–D1). Pathogenic morulae of about 0.8 µm × 0.9 µm size were visible in the cytoplasm (Figure 2C1). It was also observed that the pathogenic morulae were vacuole-shaped, which was an early manifestation (Figure 2C1–D1). Substantial purple-reddish granules were observed inside the TF-1 cells, which were visible in the cytoplasm under all cell growth stages (Figure 2B2–D2). Besides, *A. capra* morulae of 0.3 µm × 0.5 µm in size were observed in the TF-1 cytoplasm (Figure 2D2).

### 3.2. Immunocytochemistry Results

**Figure 3:**
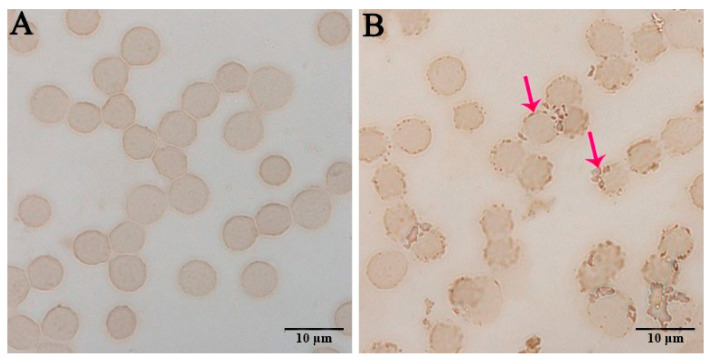
Immunocytochemistry of the erythrocytes incubated with positive goat serum: (**A**) an uninoculated human erythrocyte smear; and (**B**) the erythrocyte smear inoculated and infected with *A. capra*. The arrows indicate *A. capra*.

**Figure 4:**
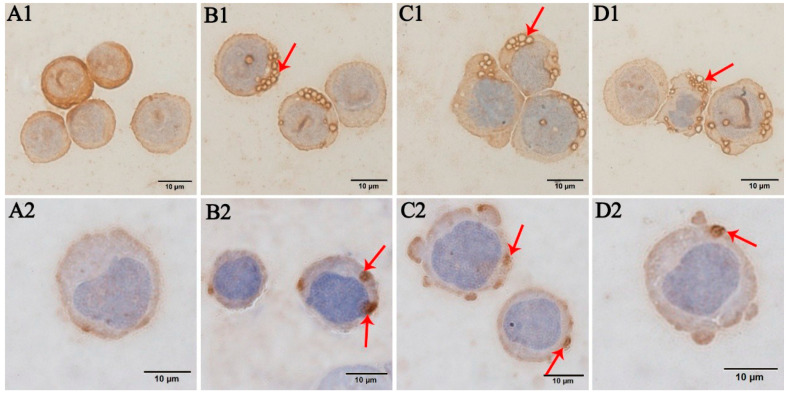
Immunocytochemistry of the HL-60 and TF-1 cells incubated with *A. capra*-positive goat serum: (**A1**,**A2**) the uninoculated cells; (**B1**–**D1**) the HL-60 cells inoculated and infected with *A. capra*, where the red arrows indicate vacuole formation in the cytoplasm of the pathogen; and (**B2**–**D2**) the TF-1 cells inoculated and infected with *A. capra*, where the red arrows indicate the morulae of the pathogen in the cytoplasm.

After 96 h of inoculation of the human erythrocytes, HL-60, and TF-1 cell smears, brown substances were visible on the surfaces of the substantially inoculated human erythrocytes (Figure 3B), indicating that these cells were infected with *A. capra*. However, this phenomenon was absent in the uninoculated human erythrocytes (Figure 3A). Substantial numbers of vacuole-like structures were observed in the cytoplasm of *A. capra*-inoculated HL-60 cells, where the vacuole edges were dark brown in color (Figure 4B1–D1). This indicated the generation of immunological reactions between the vacuoles and the antibodies. In contrast, the uninoculated HL-60 cells did not exhibit this phenomenon (Figure 4A1). Dark brown bodies were present in the cytoplasm of the *A. capra*-inoculated TF-1 cells, which were the products of reaction with antibodies (Figure 4B2–D2). However, this structure was not found in the uninoculated TF-1 cells (Figure 4A2). Thus, all three types of cells were successfully infected with *A. capra*.

### 3.3. CISH Results

**Figure 5:**
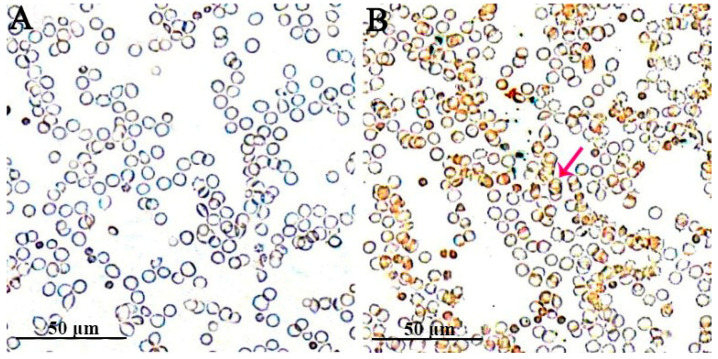
CISH analyses of the uninfected and infected human erythrocytes. The probe was designed based on the *groEL* gene sequence of *A. capra* and labeled with digoxigenin (DIG): (**A**) an uninoculated human erythrocyte smear; and (**B**) the erythrocyte smear inoculated and infected with *A. capra*. The red arrow indicates one of the *A. capra*-positive cell.

CISH analysis of the human erythrocyte smear after 96 h of inoculation revealed the presence of DAB-stained brown precipitates on the surfaces of the substantially inoculated erythrocytes (Figure 5B). This indicated that these cells were infected with *A. capra*. In contrast, this brown substance was not found in the uninoculated human erythrocytes (Figure 5A).

### 3.4. TEM Results

**Figure 6:**
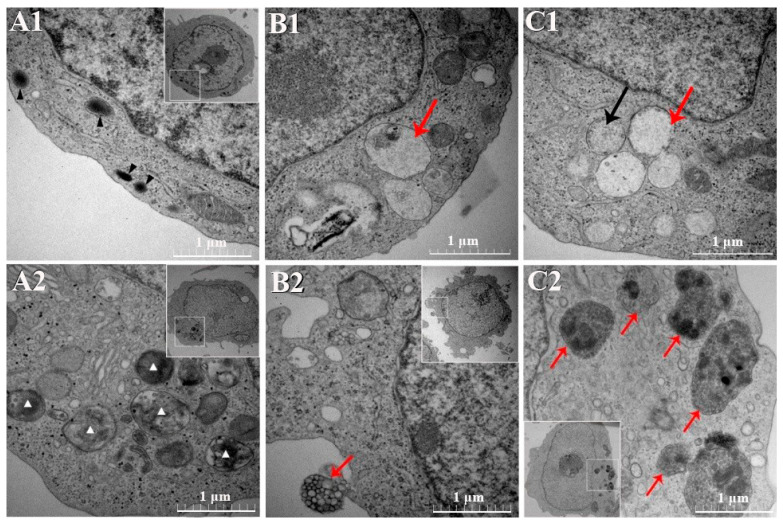
TEM photomicrographs of the HL-60 and TF-1 cells. Uranyl acetate and Reynolds’ lead citrate stain were used. (**A1**,**A2**) The negative controls. The black and white triangular symbols indicate the different states of the lysosomes. (**B1**,**C1**) The *A. capra*-positive HL-60 cells. Red arrows depict the morulae. Circular or elliptical bodies indicated by the black arrow in (**C1**) are seen inside the morulae. (**B2**,**C2**) The *A. capra*-positive TF-1 cells. Red arrows indicate the morulae.

The *A. capra*-inoculated HL-60 cells, as well as the uninoculated controls, were subjected to TEM. Multiple pathogenic morulae of about 295–518 nm diameters were observed in the cytoplasm of the HL-60 cells after 96 h of inoculation (Figure 6B1). The pathogens inside the morulae showed varying densities (Figure 6B,C1). Multiple circular or elliptical bodies were noticed inside the DC morulae (Figure 6C1). In contrast, in the uninoculated HL-60 cells, lysosomes and mitochondria were visible, while no vacuole-like inclusion structure was observed (Figure 6A1).

According to the TEM results of the inoculated and uninoculated TF-1 cells, attachment of morulae was observed outside the cell membrane of the TF-1 cells 96 h after artificial inoculation with *A. capra* (Figure 6B2). Multiple mulberry-like pathogenic morulae were visible in the cytoplasm; the largest was approximately 0.7 µm × 1 µm inside, with multiple globular bodies (Figure 6C2). The largest of these globular bodies was about 46 nm in diameter. In the uninoculated TF-1 cells, lysosomes were observed in different states, which differed distinctly from the mulberry-like morphology in the test group. Besides, the mitochondria were visible without presenting the mulberry-like structure (Figure 6A2).

## 4. Discussion

Ixodid ticks are increasing annually due to global warming, deforestation, and in-creased animal mobility. Their distribution ranges have also expanded gradually. The incidence of anaplasmosis, tick-borne disease with worldwide distribution, is recently rising [8]. *Anaplasma capra* is a novel zoonotic species that significantly increases the risk to human infections [21]. The objective of this study was to determine the infectivity of *A. capra* on human erythrocytes, HL-60, and TF-1 cells. Currently, there is no report on the artificial infection of human erythrocytes with *A. capra*. We for the first time demonstrate that the goat-derived *A. capra* can infect human erythrocytes, and TF-1 cells, evidencing its pathogenicity on human cells and zoonotic nature. Although the survival time and fecundity of the pathogen were not studied, the outcomes will be helpful for the *in vitro* cultivation of *A. capra*.

Currently, the genus *Anaplasma* is considered to comprise seven species: *A. phagocytophilum*, *A. marginale*, *A. centrale*, *A. ovis*, *A. bovis*, *A. platys*, and *A. capra* [21]. Among these, *A. phagocytophilum* infects the neutrophils in the blood of animals and humans [1,22,23]. This species can also grow on human cell lines (HL-60, THP-1, NB4, HMEC-1, and MVEC), macaque cell line (RF/6A), bovine cell line (BCE), and Ixodid tick cell lines (ISE6 and IDE8) [3,24,25]. *A. marginale* can infect ruminant erythrocytes and can be cultured in Ixodid tick cell lines, macaque cell line (RF/6A), and bovine cell line (BCE). Moreover, it can also be cultured for a short time in bovine turbinate and pulmonary artery endothelial cells as well as in *in vitro* cultured erythrocytes [18,26,27,28,29,30]. *A. ovis* has been reported to infect HL-60 cells *in vitro*; the infection can last for four months [4]. The *in vitro* cultivation of *A. centrale*, *A. bovis,* and *A. platys* have not yet been reported [31,32]. This study confirmed that the goat-derived *A. capra* can infect HL-60 and TF-1 cells. In a study by Li et al. [3] (2015), human-derived *A. capra* was demonstrated to infect HL-60 and THP-1 cells. Thus, *A. capra* from different hosts is capable of infecting human cells.

HL-60 is derived from patients with acute promyelocytic leukemia and can be differentiated into granulocytes, monocytes, or macrophages under drug induction [33]. HL-60 cells can be used to grow *A. phagocytophilum* and *A. ovis* [4,25,34]. *A. ovis* is a pathogen that obligately parasitizes erythrocytes, while *A. phagocytophilum* obligately parasitizes neutrophils. Thus, HL-60 cells have a relatively wide potential for infection with bloodborne pathogens. During the *in vitro A. marginale* infection of IDE8 cell line, the pathogen formed vacuoles to prevent acidification and avoid clearance by the lysosomal enzymes. Upon creating a synthetic deficiency of the *A. marginale* protein, the parasitic vacuoles lost their regulatory ability, began to acidify, and thus were cleared by the lysosomal enzymes [35]. In this study, the morulae of *A. capra* was also observed within the HL-60 cells through TEM and showed varying densities. They were probably the two different types (DC and RC) of the *Anaplasma* species that reflected the different developmental stages. Thus far, there has been no report related to the *in vitro* cultivation of *Anaplasma* in the TF-1 cells. 

In the TEM photomicrographs of the *A. capra*-infected TF-1 cells, the lysosomes were involved in the pathogenic vacuole formation. This might also be associated with the formation of pathogenic vacuoles and the clearing action of lysosomes. Some host cells responded to bacterial infection by phagocytosing bacteria and then digesting them inside the cells. Such a cellular defense system has been validated in several Ixodid tick cell lines (IDE12 and DAE15) [36]. When *A. phagocytophilum* isolates infected the cell lines derived from *Ixodes ricinus* and *Ixodes scapularis*, they could inhibit the apoptotic pathway of these tick cells in the early infection stages; this promoted their survival and helped their passaging in the cell lines for a long time. Besides, the pathogen infectivity might also change with its adaptability to or interaction with the host cells [25,37]. However, all of this needs to be confirmed through further research. 

## 5. Conclusions

In this study, *A. capra* isolates from goats were artificially inoculated into human erythrocytes, HL-60, and TF-1 cells that were cultured under *in vitro* conditions. Moreover, the infectivity of the pathogen in the three cell lines was confirmed. Further, the zoonotic of *A. capra* was verified. The findings of this study lay a foundation for the *in vitro* cultivation modeling of the pathogen and enable the exploration of host–pathogen interaction and pathogen invasion mechanisms. 

## Data Availability

No new data were created or analyzed in this study. Data sharing is not applicable to this article.
